# Pharmacogenetic profiles of young danish individuals with and without severe mental disorders

**DOI:** 10.1192/j.eurpsy.2021.396

**Published:** 2021-08-13

**Authors:** C. Lunenburg, J. Thirstrup, C. Gasse

**Affiliations:** 1 Department Of Clinical Medicine, Aarhus University, Aarhus, Denmark; 2 Dep. Affective Disorders, Aarhus University Hospital Psychiatry, Aarhus N, Denmark; 3 Department Of Biomedicine, Faculty of Health, Aarhus University, Aarhus, Denmark; 4 Psychosis Research Unit, Aarhus University Hospital Psychiatry, Aarhus N, Denmark; 5 Centre For Integrated Register-based Research, Aarhus University, Aarhus, Denmark

**Keywords:** genotype, phenotype, Pharmacogenetics

## Abstract

**Introduction:**

Pharmacogenetics (PGx) studies genetic variance and related differences in drug outcomes. PGx guidelines for psychotropic drugs are available (PGx drugs). By executing PGx testing in a prospective or pre-emptive setting, dose adjustments or even change of treatment type can be applied prior to start of therapy to patients who carry a specific geno- or phenotype (i.e. actionable geno- or phenotypes). By doing so, increased efficacy of therapy or reduced risk of adverse events of treatment can be accomplished. In Denmark, broad implementation of PGx is currently still low.

**Objectives:**

The aim of this study is to classify the PGx profiles of Danish individuals with and without severe mental disorders (SMD), to be used in follow-up studies investigating PGx and drug outcomes.

**Methods:**

This study made use of imputed genotyping data of the Danish iPSYCH sample, which includes 77,639 young individuals born between 1981-2005, with or without a diagnosis of one or more of five selected SMD (i.e. depression, attention-deficit/hyperactivity disorder, autism, bipolar disorder and schizophrenia). We investigated a panel of 48 genetic variants with known PGx applications (part of the U-PGx consortium, a Horizon2020 funded project on clinical relevant PGx in the EU).

**Results:**

Imputed data contains over 11 million SNPs of 77,639 individuals.

**Conclusions:**

We expect results in the end of 2020.

**Disclosure:**

We thank the iPSYCH consortium, in specific the iPSYCH PI’s (Merete Nordentoft, Anders Børglum, Preben B. Mortensen, Ole Mors, Thomas Werge and David M. Hougaard). The iPSYCH project is funded by the Lundbeck Foundation Denmark and the universities and un

